# Green-fabricated silver nanoparticles from *Quercus incana* leaf extract to control the early blight of tomatoes caused by *Alternaria solani*

**DOI:** 10.1186/s12870-024-05008-5

**Published:** 2024-04-19

**Authors:** Javaria Khatoon, Ansar Mehmood, Abd ur Rehman Khalid, Muhammad Abdul Rauf Khan, Khawaja Shafique Ahmad, Muhammad Shoaib Amjad, Urooj Bashir, Muhammad Raffi, Jarosław Proćków

**Affiliations:** 1 Department of Botany, University of Poonch Rawalakot, Rawalakot, Azad Jammu and Kashmir 12350 Pakistan; 2 Department of Plant Pathology, University of Poonch Rawalakot, Rawalakot, Azad Jammu and Kashmir 12350 Pakistan; 3 Department of Physics, University of Poonch Rawalakot, Rawalakot, Azad Jammu and Kashmir 12350 Pakistan; 4 Department of Botany, Women University of Azad Jammu & Kashmir Bagh, Bagh, 12500 Pakistan; 5 National Institute of Lasers and Optronics College, Pakistan Institute of Engineering and Applied Sciences, Nilore, Islamabad 45650 Pakistan; 6 Department of Plant Biology, Institute of Environmental Biology, Wrocław University of Environmental and Life Sciences, ul. Kożuchowska 5b, Wrocław, 51-631 Poland

**Keywords:** Silver nanoparticles, Green fabrication, Antifungal, *Alternaria solani*, Early blight of tomatoes

## Abstract

**Background:**

Early blight (EB) of Tomatoes, caused by *Alternaria solani*, is a serious fungal disease that adversely affects tomato production. Infection is characterized by dark lesions on leaves, stems, and fruits. Several agrochemicals can be used to control infection, these chemicals may disrupt environmental equilibrium. An alternative technology is needed to address this significant fungal threat. This study was designed to control the growth of EB in tomatoes caused by *A. solani*, using green-fabricated silver nanoparticles (Ag-NPs).

**Results:**

Ag-NPs were synthesized through an environmentally friendly and cost-effective approach using leaf extract of *Quercus incana* Roxb. (*Fagaceae*). The physico-chemical characterization of the Ag-NPs was conducted through UV-visible spectroscopy, scanning electron microscopy, X-ray diffraction analysis, and Fourier transform infrared spectrometry. The Ag-NPs produced were round with a mean diameter of 27 nm. The antifungal activity of these Ag-NPs was assessed through in vitro Petri plate and in vitro leaflet assays against *A. solani*. The green fabricated Ag-NPs exhibited excellent antifungal activity in vitro at a concentration of 100 mg/l against *A. solani*, inhibiting growth by 98.27 ± 1.58% and 92.79 ± 1.33% during Petri plate and leaflet assays, respectively.

**Conclusion:**

In conclusion, this study suggests the practical application of green-fabricated Ag-NPs from *Q. incana* leaf extract against *A. solani* to effectively control EB disease in tomatoes.

## Background

Tomato (*Solanum lycopersicum* L., *Solanaceae*) is a highly nutritive fruit, which some consider a vegetable, due to the large contents of different vitamins such as A, C, and other natural antioxidants, which are not found in other crops [1]. After potatoes, the tomato plant is the most profitable solanaceous vegetative crop. It is indigenous to Southern China is the leading tomato production country followed by India and United States. America and is widely refined in 140 countries around the world, with a production of 150 million tons annually [2]. Tomato production in Pakistan is crucial for both domestic consumption and export markets, playing a pivotal role in the agricultural sector’s economy due to its significant contribution to livelihoods and food security [3]. The annual production of tomatoes in Pakistan typically ranges between 1.5 million to 2.0 million metric tons, with some fluctuations from year to year but its annual production can vary due to factors such as weather conditions, agricultural practices, and government policies [4].

Several diseases caused by bacteria, viruses, and fungi are attacking tomatoes, thus reducing their yield production [5]. Among fungal diseases, the EB of tomatoes triggered by *A. solani* causes severe yield losses all over the world [6]. EB is diagnosed by the presence of dark brown or black lesions with circular rings on the leaves of potatoes and tomatoes. Other manifestations include stem lesions and fruit rot. *Alternaria* species are widespread in agricultural soils around the world [7], and they are believed to be some of the most destructive soil-borne pathogens worldwide [8].

Different chemical fungicides are used for the management of EB disease, but due to the development of resistance in mainly mutual fungal pathogens against fungicides and the risk of exposure to fungicide residues resulting in health hazards, emphasis has been placed on alternative approaches to managing *A. solani* [9]. It is vital to implement eco-friendly and environmentally safe control procedures. In this regard, nanoparticles (NPs) are seen as superior to synthetic chemicals because of their lower environmental impact. NPs have been the focus of investigators, due to their exclusive properties (for example, their shape and size range depend on their optical, electrical, and antimicrobial applications). Nanoscale objects have developed as novel antimicrobial agents due to their enormous surface area-to-volume ratio and distinctive physical and chemical characteristics that improve their interaction with bacteria and cell penetration [10–12].

NPs can be manufactured through physical and chemical methods, but the green fabrication of NPs has gained an edge over the use of physical and chemical methods due to many advantages, such as being environmentally safe, economical, and easily scaled up for large-scale production [13–15]. The former methods pose a substantial hazard to the environment and use high energy, high temperature, stress, and destructive chemicals, which are not necessary in green fabrication [16]. In the green method, use of plant extracts plays an important role in formation and stabilization of Ag-NPs. The reducing ability of plant extract helps the nucleation of Ag ions, leading to the formation of stable Ag-NPs [17]. Among the NPs, Ag-NPs have gained a lot of attention due to their possible uses in different applications [18–20]. In medicine, they are utilized for their antimicrobial properties in wound dressings, medical textiles, and antibacterial coatings on medical devices. In the field of electronics, silver nanoparticles are employed in conductive inks for printed electronics, improving conductivity and enabling flexible electronic components [21]. Additionally, in the environmental sector, they are utilized in water purification systems due to their ability to catalyze chemical reactions and remove contaminants effectively [22].

It is well accepted that Ag disrupts a wide variety of biological processes in microbes, such as the structure and functioning of cell membranes [23], [24]. The use of Ag-NPs as antibacterial agents has increased as technology (green synthesis) has made it more affordable to produce them. One of the possible uses of Ag-NPs is to control plant disease. Compared to synthetic fungicides, Ag-NPs exhibit several ways of inhibiting microorganisms [25], making them potentially safer methods of managing a variety of plant infections [26]. Previous research has shown some evidence of the potential of Ag-NPs in mitigating plant diseases [27–29]. Several inhibiting mechanisms of silver nanoparticles against microorganisms or pathogens have been reported like cell membrane and DNA damage, ROS generation and enzyme inhibition [30]. The objectives of this study were to engineer green-fabricated Ag-NPs from Quercus incana Roxb. leaf extract (Fagaceae) and to demonstrate their use in the management of *Alternaria solani*-caused tomato EB. The control measures used for managing early blight in plants, such as tomatoes and potatoes, can also be applied to other fungal diseases affecting various crops. Some of these diseases include late blight, powdery mildew and botrytis. These diseases share similar control measures with early blight due to their fungal nature and the importance of managing environmental conditions, cultural practices, and the application of appropriate fungicides to reduce disease incidence and severity. For this, in vitro petri plate and in vitro leaflet essay methods were used to gauge the antifungal efficacy of Ag-NPs against *A. solani*.

## Methods

### Chemicals

Silver nitrate (Sigma-aldrich), acetone (Sigma-aldrich), Potato Dextrose Agar (Merck), and sodium hypochlorite (Sigma-aldrich). All the chemicals were A grade.

### Green fabrication of Ag-NPs

For the green fabrication of Ag-NPs, the leaf extract of *Q. incana* was used. Leaves were picked from *Q. incana* plants grown in Phagwati Hajira, Poonch Azad Kashmir, Pakistan (33.57ºN, 73.62ºE). The plant was recognized with the help of Flora of Pakistan (http://legacy.tropicos.org/Project/Pakistan) and authenticated by World Flora Online (https://www.worldfloraonline.org). The collected leaves of *Q. incana* were washed numerous times with distillation water to remove dust, followed by drying at room temperature. The 10 g of leaves were cut into small pieces, put into 100 ml of distilled water, and boiled for 20 min on a hot plate. The solution was allowed to cool before filtering through Whatman filter paper No. 42, and the filtrate was then used to create Ag-NPs. To make green Ag-NPs, 20 ml of leaf filtrate was mixed with 80 ml of a 1 mM AgNO_3_ solution, which was kept at room temperature and the color change was monitored. Once the color of the solution had fully formed after 24 h, it was centrifuged for 15 min at 9000 rpm. The supernatant was discarded and the pellet of AgNPs was frozen dried to obtain them in powdered form. The powder of AgNPs was used for characterization and antifungal activity.

### Characterization of Ag-NPs

#### UV-visible spectroscopy

The reaction solution was subjected to UV-visible analysis within the range of 300 to 800 nm wavelength after 0 and 24 h of reaction time using a C-7200 spectrophotometer [31].

#### Scanning electron microscopy (SEM)

For SEM and EDX analysis, a small amount of Ag-NPs was placed on carbon-coated copper grid, dried under mercury lamp and examined on the MAIA3 TESCAN [31].

#### X-ray diffraction (XRD) analysis

For XRD analysis, the pure Ag-NPs were freeze dried, and then subjected to XRD [31].

#### Fourier transform infrared (FTIR) spectroscopy

For FTIR analysis, potassium bromide (KBR) was added to the freeze-dried green fabricated Ag-NPs, and the sample was examined using FTIR (SHIMADZU, IR-Prestige-21, Japan) in a spectrum range of 400–4000 cm^− 1^ with a transmittance mode of 4 cm^− 1^ resolution [31].

### Antifungal activity of green-fabricated Ag-NPs

#### Isolation and identification of fungal pathogen

**Fig. 1:**
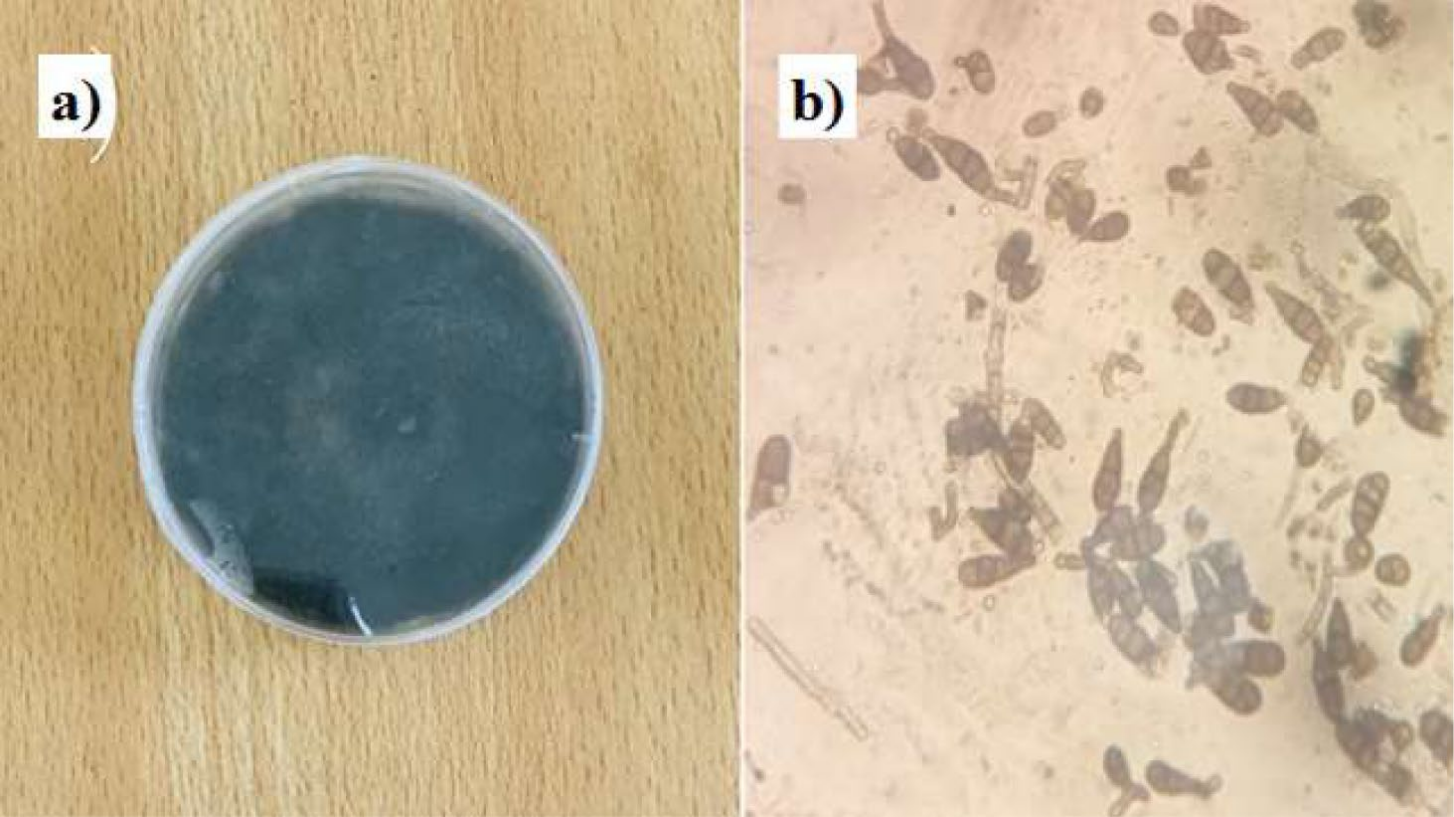
Isolation of *A. solani*, (**a**) growth in PDA and (**b**) microscopic identification

According to Katan et al. [32] identified the fungal pathogen *A. solani*, responsible for Early Blight (EB) in tomatoes, within an infected tomato fruit. The pathogen was identified based on morphological characteristics using light microscope based on several parameter such as colony behavior, pigment color of the colony, size and shape of the spore [33]. *A. solani* fungus was cultured by inoculating a potato dextrose agar (PDA) medium with spores from infected plant tissue, followed by an incubation period at optimal temperature and humidity. Isolation was involved collection of infected plant material, which was washed to remove debris, and was surface sterilizing tissue sections were transferred onto selective agar media for fungal growth. *A. solani* was cultured on PDA medium to cause sporulation (Fig. 1). The PDA was placed in the laminar flow unit after being poured into Petri plates. The infected plant sections such as leaf, fruits or stem was cultured on PDA to allow pathogen’s growth and kept at 27 ± 2 °C for 5–7 days. A conidial suspension was prepared, according to Boedo et al. [34]. Using a hemocytometer, the spore concentration was determined and corrected to 10^6^ spores/ml. Cultural characteristics were identified based on standard morphological basis including mycelial color (top plate), colony behavior (growth pattern, margin, type of colony), pigment color (bottom plate) and spore color, shape and size [35]. Cultural characteristics such as colony color, shape, and margin were recorded by visual observation of fully covered *A. solani* Petri plates on a PDA incubated at 27 ± 2°C. These isolates were subcultured until pure colonies of *A. solani* were obtained [36].

#### Pathogenicity test

**Fig. 2:**
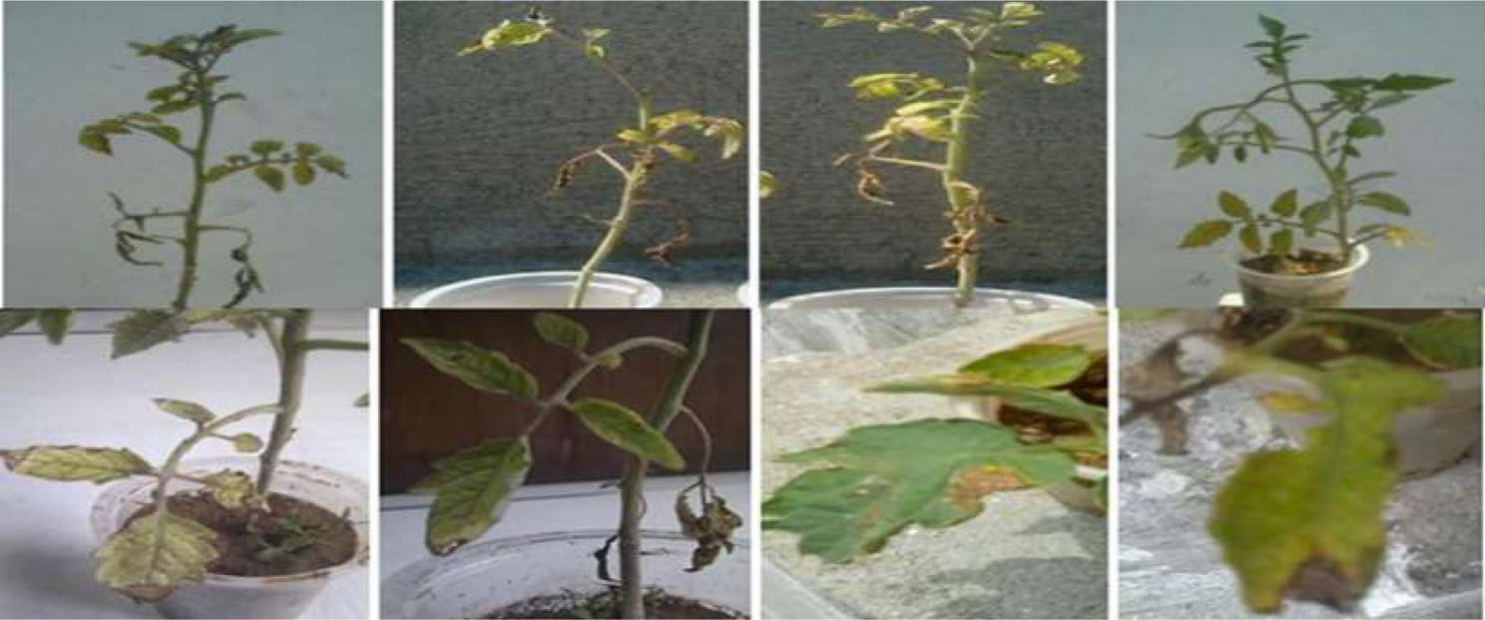
Tomato plants showing symptoms of EB after inoculation of *A. solani*

For the *pathogenicity* test, 3–5 tomato seedl were sown in plastic pots with sterilized soil. A spore suspension of 2 × 10^4^ spores/ml was made using a hemocytometer. Four weeks after for seedling development, *A. solani* suspensions were sprayed on tomato leaves while the control pots were not inoculated. These pots were placed at 27 ± 2ºC. After one week of spraying, symptoms of EB started to appear (Fig. 2). After the appearance of disease symptoms, the pathogen was re-isolated, and confirmation of the fungus was done to fulfill Koch’s postulates (microorganisms should be isolated from diseases organism and should cause diseases when introduced into a healthy organism).

#### In vitro Petri plate assay

An in vitro evaluation of green-fabricated Ag-NPs was performed against *A. solani* on a PDA medium. Different dilutions of Ag-NPs (25, 50 and 100 mg/l), AgNO_3_ solution (1 mM) and mancozeb fungicide (2 g/l) were used. The PDA medium and different treatments (1 ml) were poured into 90 × 15 mm Petri plates and incubated for 48 h. On control plates, no treatment was given. Following incubation, 7 mm diameter agar plugs containing fungus were injected concurrently in the middle of each Petri dish and allowed to incubate at 27 ± 2ºC. Following an incubation of two weeks, the radial expansion of the colony was measured. The test was repeated three times to ensure the data consistency. The percentage of inhibition (%) was calculated using the following formula:

$$\text{I}\text{n}\text{h}\text{i}\text{b}\text{i}\text{t}\text{i}\text{o}\text{n }    \text{r}\text{a}\text{t}\text{e} \left(\text{\%}\right) = \frac{\text{C}-\text{T}}{\text{C}}\times 100$$

where C is the radial growth of the fungal pathogen in the control plate and T is the radial growth of the fungal pathogen in Ag-NP-treated plates.

#### In vitro leaflet assay

The healthy seedlings of tomato (4 weeks old) were removed from the soil and washed with tap water to remove any soil debris. The roots were then dipped in a solution containing A. solani for 30 min to stimulate disease infection. These seedlings were then planted in pots under the greenhouse and left for 2 weeks. During this, various intercultural operations such as sheltering, irrigation, gap filling, weeding, and insecticide spraying were performed. After 15 days of plantation, Ag-NPs (25, 50, and 100 mg/l), AgNO_3_ (1 mM), mancozeb fungicide (2 g/l) and distilled water (control), were sprayed twice at a 15-day interval. The four-week-old healthy tomato seedlings were uprooted, and the roots were gently washed with slow-flow tap water to remove peat debris. The roots of the tomatoes were then immersed in a conidia suspension of *A. solani* for 30 min for artificial inoculation. The seedlings were transferred to pots and left for seven days. One seedling was planted in a separate pot. After transplanting the 15-day seedlings, various intercultural operations such as sheltering, irrigation, gap filling, weeding, and insecticide spraying were performed. The experiment was carried out under artificially inoculated conditions. All treatments, including Ag-NPs (25, 50, and 100 mg/l), AgNO_3_ (1 mM), mancozeb fungicide (2 g/l) and distilled water (control), were sprayed twice at a 15-day interval, starting 15 days after transplantation. Precautions were taken to prevent the spray from drifting to other pots by using a barrier made of polythene. For recording data on leaf infection, five plants were randomly selected from each treatment. The diseased area on the leaf surface was measured and the inhibition rate was measured using the following equation:

$$\text{I}\text{n}\text{h}\text{i}\text{b}\text{i}\text{t}\text{i}\text{o}\text{n } \text{r}\text{a}\text{t}\text{e} \left(\text{\%}\right)= \frac{\text{C}-\text{T}}{\text{C}}\times 100$$

where C is the diseased area of the control leaves and T is the diseased area of the treated leaves.

#### Statistical analysis

Antifungal experiments were performed in three replicates. Data were statistically analyzed (one way ANOVA) using a statistical software named SPSS 16.0. The *p-value* less than 0.05 was considered a significant result.

## Results and discussion

### Green fabrication of Ag-NPs

**Fig. 3:**
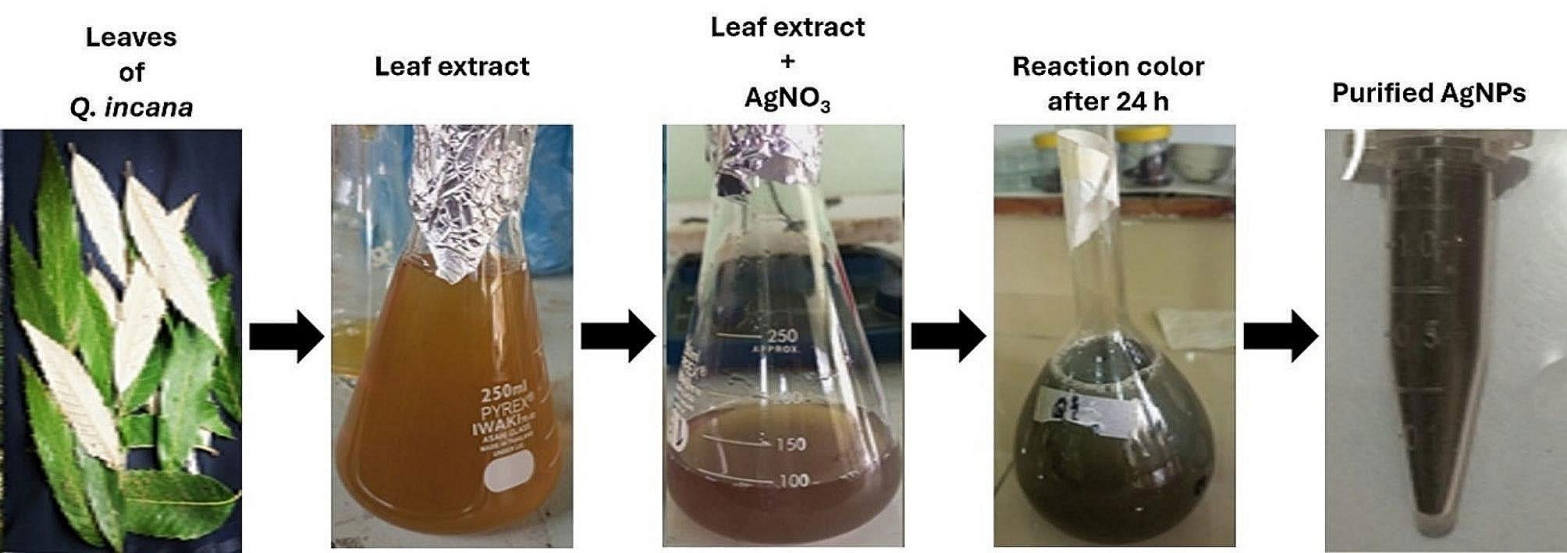
Green fabrication of Ag-NPs, (**a**) leaves of *Q. incana*, (**b**) leaf extract, (**c**) reaction solution (leaf extract + AgNO_3_), (**d**) reaction solution (leaf extract + AgNO_3_) after 24 h, and (**e**) purified Ag-NPs

The fabrication of Ag-NPs in the colloidal solution of leaf extract and AgNO_3_ was initially determined by color change. The pure solution of AgNO_3_ was virtually transparent and the color of the leaf filtrate was light brown. When the leaf filtrate and AgNO_3_ were mixed, the solution acquired a brown color. After 24 h of reaction, the color of the mixture developed to dark brown, suggesting the fabrication of Ag-NPs in the solution (Fig. 3). Ag-NPs have the distinct quality of turning the solution into a dark brown hue [37]. These color variations are carried out by electron oscillation and are also related to the surface plasmon resonance (SPR) of the accumulated Ag-NPs [38].

### UV-Visible spectroscopy

**Fig. 4:**
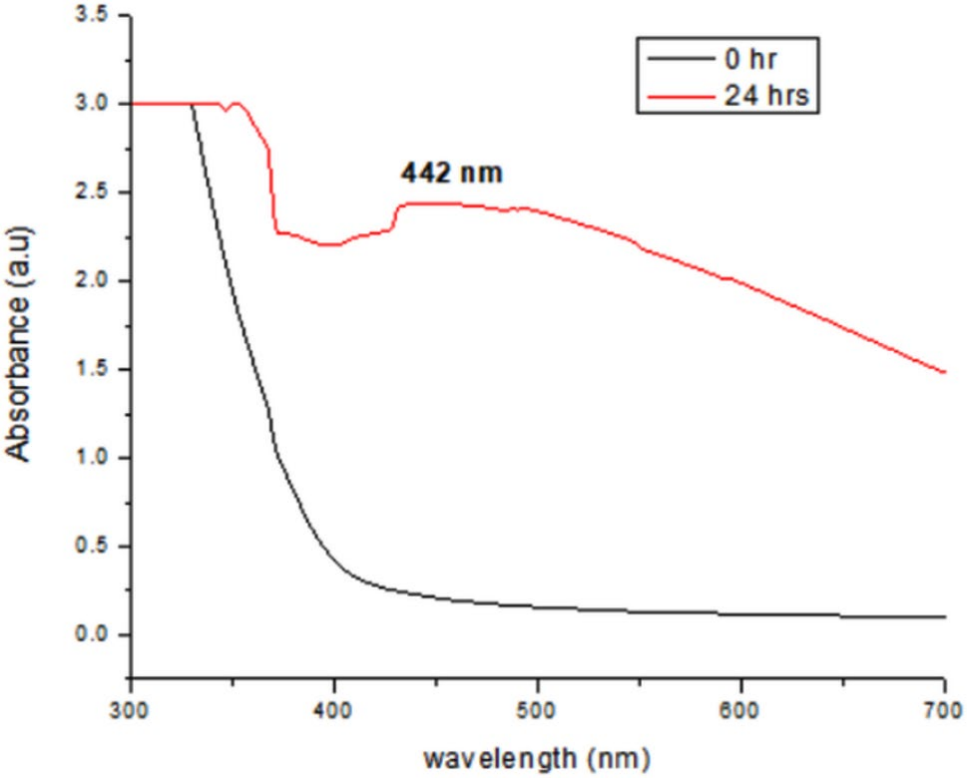
UV-visible spectrum of the reaction solution carrying Ag-NPs made from leaf extract of *Q. incana*

After the development of the color, the reaction solution was subjected to ultraviolet-visible (UV-vis) spectroscopy for other evidence of the fabrication of Ag-NPs. A clear absorption band was observed at 442 nm due to the SPR verifying the green fabrication of Ag-NPs (Fig. 4). The primary absorption peak observed at 442 nm indicates the presence of Ag-NPs, as evidenced by numerous studies documenting absorption peaks of Ag-NPs typically falling within the range of 400 to 500 nm [39–42].

### SEM and EDX analysis of Ag-NPs

**Fig. 5:**
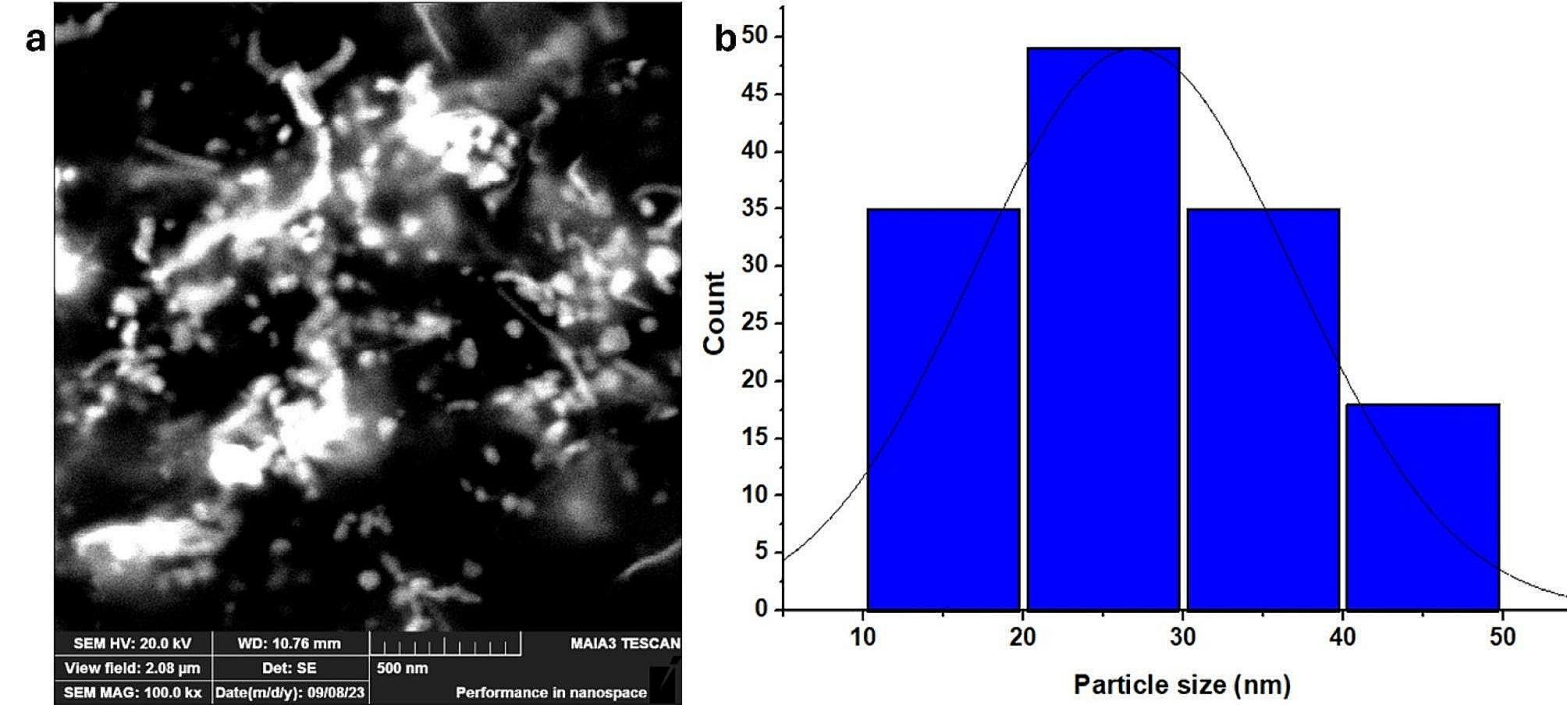
Morphological envision of Ag-NPs, (**a**) SEM image, and (**b**) particle size distribution

**Fig. 6:**
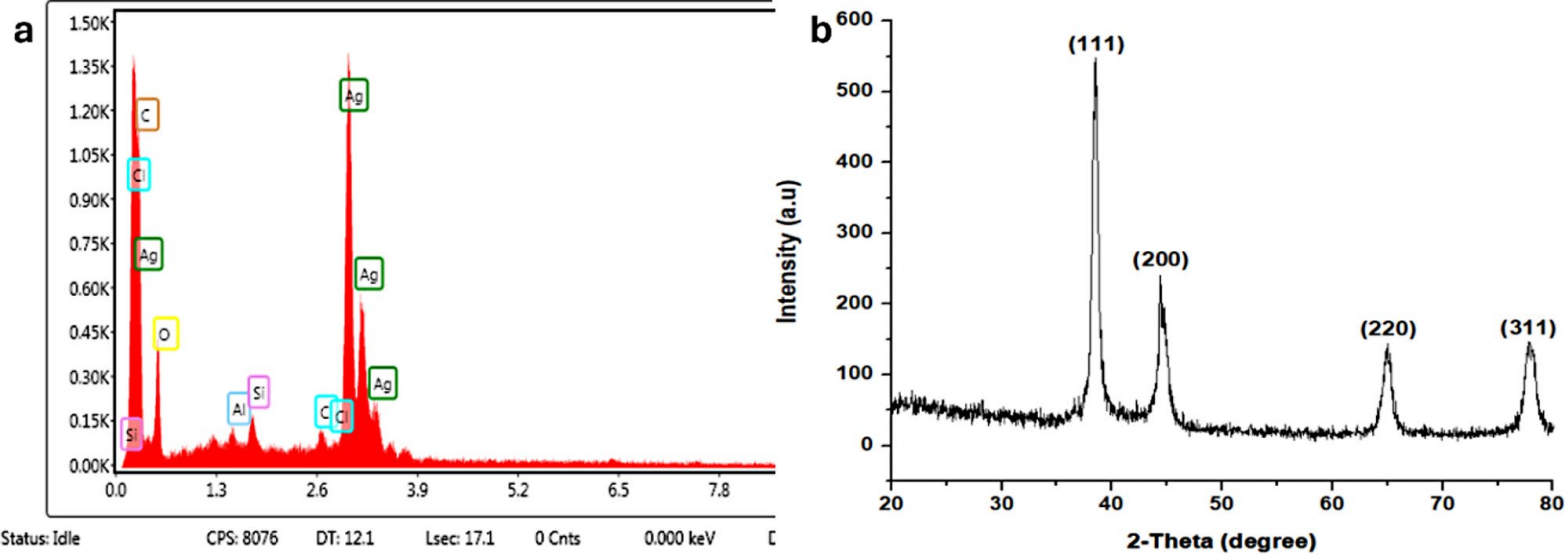
EDX spectrum of Ag-NPs (**a**) and (**b**) XRD pattern of Ag-NPs

The SEM image of green-fabricated Ag-NPs from *Q. incana* confirmed the existence of round NPs in the sample. The mean diameter of Ag-NPs was 27 nm, calculated from the SEM image through Image J software (Fig. 5). The EDX analysis of Ag-NPs is shown in Fig. 6a. The EDX image showed a convincing signal peak for Ag metal at 2.8 keV, confirming the presence of Ag-NPs. Multiple studies corroborate these findings, indicating that the Ag metal exhibits a prominent peak at 2.8 keV in the EDX spectrum. Additionally, weak peaks corresponding to other inorganic ions like Si, Cl, and Al were observed, likely stemming from the bioorganic constituents of the extracts. The analysis also reveals significant amounts of K, Si, Cl, and Al [43, 44].

### XRD analysis of green-fabricated Ag-NPs

The XRD pattern indicated 4 intense peaks at 38.84˚, 44.94˚, 64.84˚, and 78.11˚, corresponding to (111), (200), (220), and (311), respectively, indexing the face-centered cubic structure of Ag-NPs (Fig. 6b). The discovered lattice planes (111), (200), (220), and (311), in accordance with JCPDS file no. 04-0783 of silver and demonstrated face centered cubic structure of Ag-NPs [45]. The strong and narrow diffraction peaks also indicate pure and crystalline Ag-NPs [46]. The nanoparticles are in the nano range, as shown by the sharp peaks [47].

### Fourier transform infrared (FTIR) spectroscopy

**Fig. 7:**
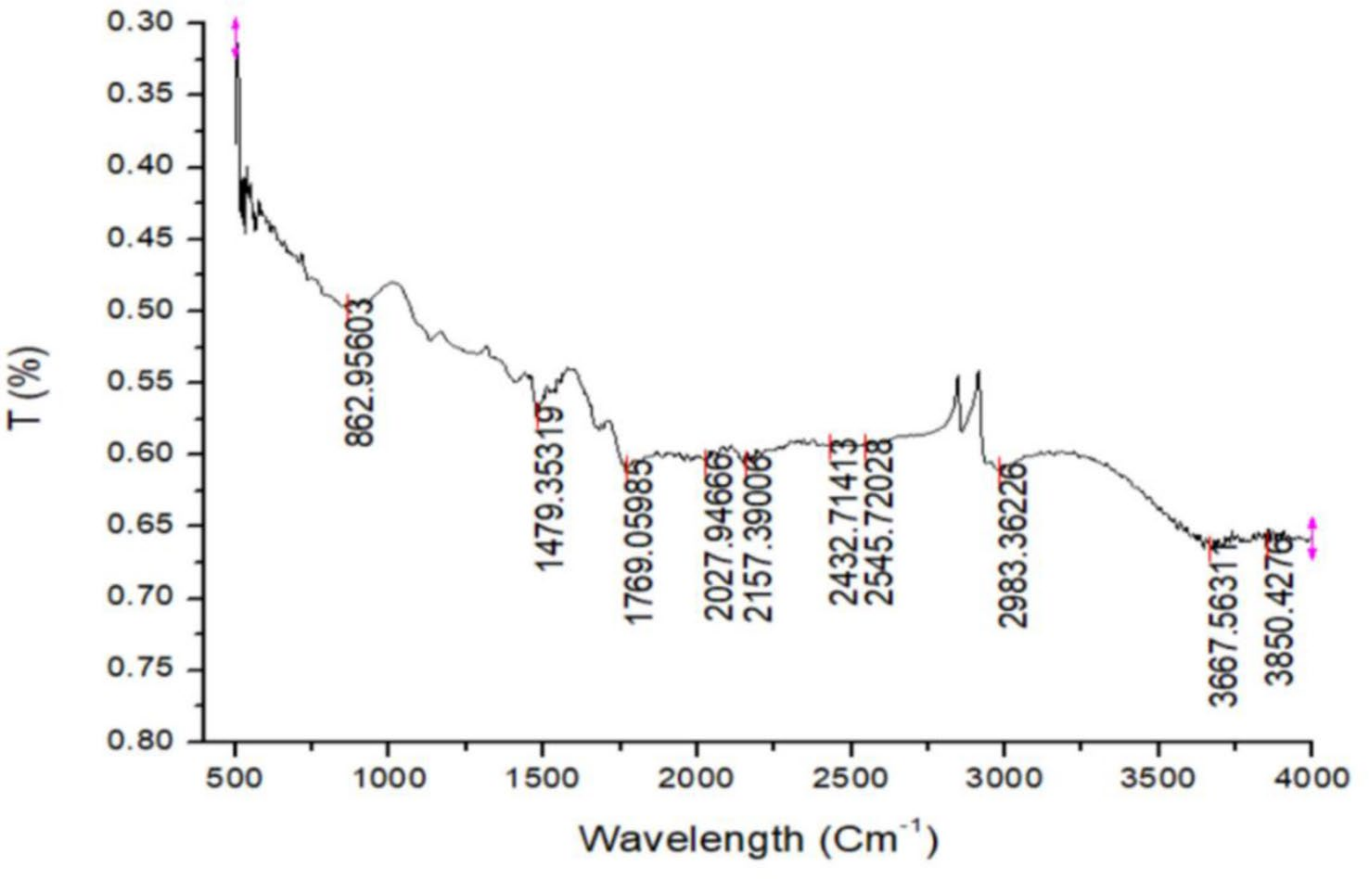
FTIR spectrum of green-fabricated Ag-NPs

The chemicals and metabolites acting as reducing and capping agents of Ag-NPs are determined by the FTIR of Ag-NPs. Compound functional groups were suggested by stretching and bending vibrations in the 4000 –500 cm^− 1^ region [48]. Figure 7 illustrates the FTIR spectrum of Ag-NPs. The bands at 3667 and 2545 cm^− 1^ are assigned to the O-H stretching of H-bonded alcohols and phenols. The peak formed at 2157 cm^− 1^ corresponded to the functional group N = N = N stretching of the N-bonded azide. The peak at 2027 cm^− 1^ is consigned to the functional group N = C = S stretching due to isothiocyanate. The other peaks in the spectrum at 2983, 1769, 1479, and 862 cm^− 1^ could be ethers, esters, polyphenols, or aromatic compounds. These key functional groups, which include N-H, C-N, and C-O, are found in pigments, proteins, amino acids, and flavonoids. They may also participate in the bioreduction process of Ag by plant extract [49].

### Antifungal activity of Ag-NPs against *A. solani* by Petri plate essay

**Fig. 8:**
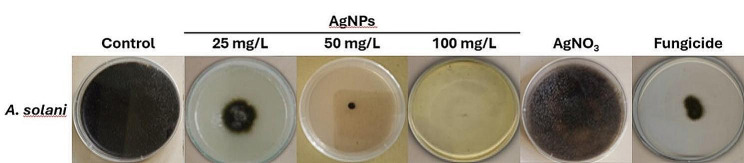
Antifungal activity of Ag-NPs in vitro (Petri plate essay) against *A. solani*

Ag-NPs significantly reduced the radial growth and increased the inhibition rate of *A. solani* compared to the control (Fig. 8). The percentage of inhibition increased gradually as the amount of Ag-NPs increased from 25 to 100 mg/l (Table 1). However, the highest inhibition rate was produced by 100 mg/l Ag-NPs (98.27 ± 1.58%). The fungicide inhibited *A. solani* growth by 80.39 ± 0.33% of the inhibition rate. Ag was also used in ionic form (AgNO_3_) to find if the antifungal activity was due to Ag ions or Ag-NPs. The results showed that AgNO_3_ did not inhibit the growth of *A. solani*. Previous studies also stated the antifungal potential of Ag-NPs versus *A. solani*. For example, Ag-NPs from different sources effectively inhibited *A. solani* growth [50, 51].

**Table 1:** Antifungal activity (Petri plate assay) of Ag-NPs fabricated from *Q. incana* against *A. solani*

Concentration	Control (0%)	Silver nanoparticles			AgNO_3_ (1 mM)	Mancozeb (2 g/l)
		25 mg/l	50 mg/l	100 mg/l		
Colony diameter (mm)	86.67 ± 2.22 ^a^	22.00 ± 1.58 ^b^	04.66 ± 1.67 ^d^	1.50 ± 2.33 ^e^	85.33 ± 1.67 ^a^	17.00 ± 0.67 ^c^
MGI (%)	0	74.02 ± 0.58 ^d^	94.62 ± 0.67 ^b^	98.27 ± 1.58 ^a^	01.55 ± 0.67 ^e^	80.39 ± 0.33 ^c^

Values are the means of three replicates and the standard deviation followed by the same letters in a row. They are significantly different at *p* = 0.05 by the LSD test

### Antifungal activity of Ag-NPs against *A. solani* by leaflet assay

**Fig. 9:**
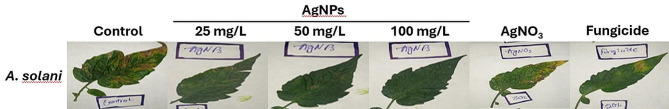
Antifungal activity in vitro (leaflet essay) of Ag-NPs against *A. solani*

In a leaflet assay, 1 month old tomato leaves infected with *A. solani* were sprayed with various dilutions of Ag-NPs (25, 50, and 100 mg/l), AgNO_3_, and fungicide in a greenhouse. Ag-NP and fungicides were discovered to successfully stop the growth and spread of *A. solani* on tomato leaves (Fig. 9). Although AgNO_3_ did not effectively stop the fungus from spreading on tomato leaves, the results are shown in Table 2. The highest inhibition rate was shown by 100 mg/l Ag-NPs, with an inhibition rate of 92.79 ± 1.33%. The fungicide reduced the spread of *A. solani* on tomato leaves by 67.80 ± 0.67%.

**Table 2:** Antifungal activity (leaflet assay) of Ag-NPs fabricated from *Q. incana* against *A. solani*

Concentration	Control (0%)	Silver nanoparticles			AgNO_3_ (1 mM)	Mancozeb (2 g/l)
		25 mg/l	50 mg/l	100 mg/l		
Diseased area (mm)	78.67 ± 1.58 ^a^	26.33 ± 1.84 ^b^	21.33 ± 2.38 ^c^	05.67 ± 2.09 ^d^	76.33 ± 2.91 ^a^	25.33 ± 1.33^b^
% of inhibition	0	66.53 ± 1.33 ^c^	72.89 ± 1.58 ^b^	92.79 ± 1.33 ^a^	02.89 ± 1.44 ^d^	67.80 ± 0.67 ^c^

Values are the means of 3 replicates ± standard deviation followed by different letters in a row that are significantly different at *p* = 0.05 by the LSD test

In general, our results clearly demonstrate that Ag-NPs could effectively control the EB of tomatoes caused by *A. solani*. These results also recommend that Ag-NPs may be used as prospective agrochemicals in the future to manage plant disease [52]. Many other studies have shown that Ag-NPs are effective in controlling pepper anthracnose disease with a concentration of 100 ppm in both in vitro and in vivo evaluations [27]. Similarly, Ag-NPs successfully controlled *Ralstonia solanacearum* fungal disease [53], and chitosan and chitosan-meal nanocomposites were used as nano-agrochemicals against chickpea fusarium wilt [54]. The biosynthesized Ag-NPs by chickpea rhizospheric microflora control wilt disease [55], while chitosan NPs show antifungal properties against pathogens, including *A. solani*, in tomato under in vivo conditions [56]. Pathogens’ ability to replicate their DNA is likely hindered by Ag-NPs, which also deactivate cellular proteins and enzymes [57]. Ag-NPs fight collateral damage, prevent plants from becoming infected, and block pathogen growth [58]. Phenolic compounds are the primary components of many anti-pathogenic chemicals produced by plants, which function as the primary line of defense. The formation of phenols in plants is stimulated by Ag-NPs, helping to reduce the severity of disease [59]. Higher resistant plants are better equipped to reduce the formation of reactive oxygen species after a pathogen attack, reducing stress enzyme activity in plants treated with Ag-NPs [60].

## Conclusion

In this study, spherical Ag-NPs with an average size of 27 nm were successfully synthesized in an environmentally friendly manner from the leaf extract of *Q. incana*. These green-fabricated Ag-NPs showed excellent antifungal activity against EB causing *A. solani* both in the Petri plate and leaflet assays. The results of this research clearly suggest the use of Ag-NPs as an effective nanofungicide against EB of tomato disease. As an antifungal agent, the use of green-fabricated Ag-NPs can reduce the danger of toxicity and environmental contamination that comes with the use of chemical fungicides.

## Data Availability

All data generated or analyzed during this study are included in this published article.
